# Effectiveness of DASH Diet versus Other Diet Modalities in Patients with Metabolic Syndrome: A Systematic Review and Meta-Analysis

**DOI:** 10.3390/nu16183054

**Published:** 2024-09-10

**Authors:** Juan José Valenzuela-Fuenzalida, Vicente Silva Bravo, Laura Moyano Valarezo, María Fernanda Delgado Retamal, Josefa Matta Leiva, Alejandro Bruna-Mejías, Pablo Nova-Baeza, Mathias Orellana-Donoso, Alejandra Suazo-Santibañez, Gustavo Oyanedel-Amaro, Hector Gutierrez-Espinoza

**Affiliations:** 1 Departamento de Morfología, Facultad de Medicina, Universidad Andrés Bello, Santiago 7591538, Chile; juan.kine.2015@gmail.com (J.J.V.-F.); vicentesb2003@gmail.com (V.S.B.); ldmoyanov@gmail.com (L.M.V.); fernanda.delgado1771@gmail.com (M.F.D.R.); mattajosefa@gmail.com (J.M.L.); alejandro.bruna@upla.cl (A.B.-M.); pablo.nova@usach.cl (P.N.-B.); mathor94@gmail.com (M.O.-D.); 2 Departamento de Ciencias Química y Biológicas, Facultad de Ciencias de la Salud, Universidad Bernardo O’Higgins, Santiago 8370993, Chile; 3 Escuela de Medicina, Universidad Finis Terrae, Santiago 7501015, Chile; 4 Department of Morphology and Function, Faculty of Health and Social Sciences, Universidad de Las Americas, Santiago 8370040, Chile; alej.suazo@gmail.com; 5 Facultad de Ciencias de la Salud, Universidad Autónoma de Chile, Santiago 8910060, Chile; g.oyanedelamaro@gmail.com; 6 One Health Research Group, Universidad de Las Americas, Quito 170124, Ecuador

**Keywords:** metabolic syndrome, X syndrome, dash dietary, DASH diet

## Abstract

Background: Metabolic syndrome refers to the coexistence of several known cardiovascular risk factors, including insulin resistance, obesity, atherogenic dyslipidemia, and hypertension. These conditions are interrelated and share underlying mediators, mechanisms, and pathways. Improvement in dietary habits has been shown to improve metabolic parameters in patients undergoing treatment with different diets. Methods: A systematic search in different databases was realized using the keywords “Metabolic syndrome”, “X syndrome”, “Dash dietary” and “Dash diet”. Finally, six studies were included in this meta-analysis. Results: All articles comparing the DASH diet vs. other diet modalities reported significant differences in favor of the DASH diet on Systolic blood pressure (SBP) (standardized mean difference [SMD] = −8.06, confidence interval [CI] = −9.89 to −7.32, and *p* < 0.00001), Diastolic blood pressure (SMD = −6.38, CI = −7.62 to −5.14, and *p* < 0.00001), Cholesterol HDL (SMD = 0.70, CI = 0.53 to 0.88, and *p* < 0.00001) and Cholesterol LDL (SMD = −1.29, CI = −1.73 to −0.85, and *p* < 0.00001) scales. Conclusions: The DASH diet has been shown to be beneficial in altered parameters in patients with MS, and the resulting improvements can significantly affect the daily health of these patients. We therefore recommend that professionals who manage these pathologies promote the use of the DASH diet for the management of specific symptoms.

## 1. Introduction

Metabolic syndrome refers to the coexistence of several known cardiovascular risk factors, including insulin resistance, obesity, atherogenic dyslipidemia, and hypertension. These conditions are interrelated and share underlying mediators, mechanisms, and pathways. Improvement in dietary habits has been shown to improve metabolic parameters in patients undergoing treatment with different diets. Dietary approaches to stop hypertension, better known as the DASH diet, is a dietary regimen that was created to reduce high blood pressure based on the patient’s diet. It is mainly characterized by focusing on the consumption of lean proteins, lots of fruits, whole grains or complex carbohydrates, low-fat dairy products, and foods low in unsaturated fats as well as reduced consumption of red meat and added sodium, preferring the consumption of fish, nuts, and vegetables instead, as reported by Hashemi et al. [1], who also indicated its usefulness in reducing diastolic blood pressure.

Although its origin lies in combating high blood pressure, to date, the evidence shows that it also presents favorable effects in patients who suffer from type 2 diabetes mellitus, metabolic syndrome, and dyslipidemia, thus offering in the first instance an effective treatment alternative without drugs, which could generate better adherence in those patients who cannot or prefer not to take drugs.

An example of this fact is the report by Lien et al. [2] that the DASH diet, in addition to improving systolic blood pressure, can reduce diastolic blood pressure, total cholesterol, HOMA, and fasting insulin among people with and without metabolic syndrome. This is because the possible mechanisms by which DASH lowers BP come from studies of vascular and endothelial function, since this diet encourages a lower intake of saturated fats. As Lien et al. explain, the consumption of fatty foods can affect endothelial function and oxidative stress, and endothelial dysfunction is also related to high blood pressure.

Various studies support the set of foods on which the DASH diet focuses. For example, Paula et al. [3] showed that increasing potassium consumption helps to reduce blood pressure and also has beneficial effects on blood lipids, insulin resistance, and the risk of coronary heart disease, in addition to reducing the components of metabolic disease and reducing the calculated risk of cardiovascular diseases and diabetes. Additionally, Zou et al. [4] demonstrated that the DASH diet, being rich in anti-inflammatory factors (including unsaturated fatty acids, vitamin C, magnesium, and dietary fiber), can reduce the expression of serum inflammatory factors such as IL-6 and CRP, delaying and improving atherosclerosis, since reductions in total cholesterol, LDL-C, HDL-C, and apolipoprotein a1 levels have been demonstrated.

Another area in which this diet is useful and offers significant improvement is in pregnant women who have gestational diabetes. Asemi’s study [5] in Iran showed an improvement not only in terms of insulin sensitivity and metabolic pathways in the fasting and postprandial states but also in the side effects that diabetes generates in a fetus, reducing the rate of fetal macrosomia and complications that lead to having to perform a cesarean section. Just as the mothers who followed this nutritional regimen showed greater speed in recovering normal glucose values after childbirth, the neonates of fathers who followed this diet presented a lower body mass than those who did not. This meal plan is a treatment option that places less stress on the mother than insulin injections or taking medications to increase insulin sensitivity, but it maintains control over blood glucose levels and improves the health of both the mother and the fetus.

Drugs to treat type 2 diabetes, high blood pressure, metabolic syndrome, and dyslipidemia usually carry side effects that can quickly generate other pathologies with prolonged use or overload the liver. Accordingly, the DASH diet could become a non-invasive way to treat these problems at the same time by helping to control weight, increasing insulin sensitivity, reducing systolic blood pressure, reducing triglycerides, and increasing the presence of H-LDL cholesterol, which leads to a decrease in V-LDL cholesterol. This form of treatment also has the advantage that, statistically, both men and women respond similarly regardless of the patient’s weight or syndrome, which provides a more uniform method of analyzing the effect it will have on the patient. In addition, as it is a therapy based on a lifestyle change, it is applicable in patients from pediatric to geriatric stages, without excluding pregnant women or causing harm to gestating fetuses.

On the other hand, metabolic syndrome does not refer to a singular disease, but rather to a set of factors that predispose individuals to the future development of metabolic conditions, such as type 2 diabetes mellitus and high blood pressure, or both together. The diagnosis of this syndrome focuses on five main criteria: abdominal obesity, a high level of triglycerides, a low amount of HDL cholesterol in the blood, high blood pressure, and a high level of fasting glucose. With three or more of these criteria, a person is considered to have this syndrome.

Of all these factors, the DASH diet helps mitigate the circumstances that generate the tendency to generate diseases in metabolic functioning. A low amount of HDL cholesterol increases the possibility of coronary heart disease and the accumulation of fatty plaques in the arteries that lead to an increase in blood pressure. In turn, this leads to an increase in blood glucose levels sustained over time, leading to dyslipidemia in the level of triglycerides. Moreover, it should not be ruled out that obesity is a constant risk factor in various diseases. Throughout the following study, we will analyze the DASH diet’s effectiveness in helping reduce the evolution of metabolic syndrome in comparison to other diets.

In our study, we compare the DASH diet with other diet modalities, which include psychological or therapeutic support, education regarding the consumption of appropriate foods, exercise, different diets, groups with neutral diets, or the consumption of placebos. All this is to study in depth the DASH diet’s effectiveness in reducing factors contributing to the development of metabolic syndrome or in patients who already have it to evaluate whether they present an improvement, given that studies such as that of Saslow et al. [6] suggest that the DASH diet may require the integration of psychological support to be able to clinically reduce blood pressure.

The objective of this review is to determine the effectiveness of using the DASH diet versus other types of diets for patients with metabolic syndrome.

## 2. Methods

### 2.1. Protocol and Registration

This systematic review and meta-analysis were performed and reported according to the Preferred Reporting Items for Systematic Reviews and Meta-Analyses (PRISMA) statement [7] PROSPERO ID: CRD42024561532.

### 2.2. Literature Search

We systematically searched electronic databases for the literature search, including MEDLINE (via PubMed), EMBASE, SCOPUS, the Cochrane Central Register of Controlled Trials, the Cumulative Index to Nursing and Allied Health Literature, and Web of Science, covering records from the earliest time to June 2024. Randomized or controlled clinical trials that were published in English or Spanish were included. The following keywords were used in different combinations: “DASH diet”, “DASH therapy”, “DASH food”, and “metabolic syndrome”. Two authors (JJV-F and VS-C) independently screened the titles and abstracts of the references retrieved from the searches. We obtained the full text for references that either author considered to be potentially relevant. We involved a third reviewer (MB-V) if a consensus could not be reached (Supplementary Table S1).

### 2.3. Study Selection

For the studies included in this meta-analysis, the following inclusion criteria were adopted: patients with symptoms that were directly associated with metabolic syndrome and patients who were administered the DASH diet at different doses. The outcomes to be evaluated were pain, disability, symptom improvement, and quality of life scales; the types of studies included were clinical trials, randomized clinical trials, and experimental studies. Studies with the following characteristics were excluded: letters to the editor, reports/case series, reviews or non-human trials, studies that enrolled patients with other diseases, and studies that administered therapies other than the DASH diet or did not have a control group as a comparator.

### 2.4. Data Extraction and Quality Assessment

For this item, two authors from the research team (LM-V and VS-B) independently analyzed the relevant data for each trial, namely the articles’ authors and year of publication, type of study and total number of participants, statistical values and main results, geographical region, gender distribution, intervention dose, and type of administration. The methodological quality of the included studies was assessed using the Cochrane Risk of Bias (RoB, Cochrane collaboration, version 13.1.6) [8]. This tool assesses RoB in 7 domains: generation of a randomized sequence, concealment of the randomization sequence, blinding of participants and treatments, blinding of outcome assessment, incomplete results, selective reporting of results, and other sources of bias. Each domain could be considered to have a “low”, “unclear”, or “high” RoB. Disagreements were resolved by discussion or determined by a third reviewer (JJV-F) if consensus could not be reached. The agreement rate between reviewers was calculated using kappa statistics, resulting in substantial agreement with a value of 0.88.

### 2.5. Data Synthesis and Analysis

To assess the DASH diet’s effect, various scales were used: systolic pressure, diastolic pressure, HDL cholesterol, and VLDL cholesterol. These scales were analyzed as continuous outcomes. The effect size was calculated as the standard mean difference (SMD). We calculated the SMD score using Cohen’s d as the effect size statistic, categorizing the effect sizes as trivial (<0.2), small (0.2–0.5), medium (0.6–0.8), or large (>0.8). Additionally, depending on the heterogeneity of the data, the Hartung–Knapp–SidikJonkman random-effect or Mantel–Haenszel fixed-effect method was used to quantify the pooled effect size of the studies included. We presented the effect sizes as SMD, with their respective 95% confidence intervals (CIs) in the range between 2 and −2. The results’ heterogeneity was evaluated using the *I*^2^ statistic, which considers 0% to 40% as “may not be important”, 30% to 60% as “moderate”, 50% to 90% as “substantial”, and 75% to 100% as “considerable” heterogeneity. Furthermore, we conducted a visual inspection to detect overlapping CIs in the forest plots as well as the corresponding *p*-values. The meta-analysis was performed using RevMan 5.4.

### 2.6. Rating the Quality of Evidence

The synthesis and quality of evidence for each outcome were evaluated using the grading of recommendation, assessment, development, and evaluation (GRADE). The quality of the evidence was classified into 4 categories: high, moderate, low, and very low [9]. We used the GRADE profiler to import the data from RevMan 5.4 to create a “summary of findings” table, which can be found in Supplementary Table S3.

## 3. Results

### 3.1. Study Selection

**Figure 1:**
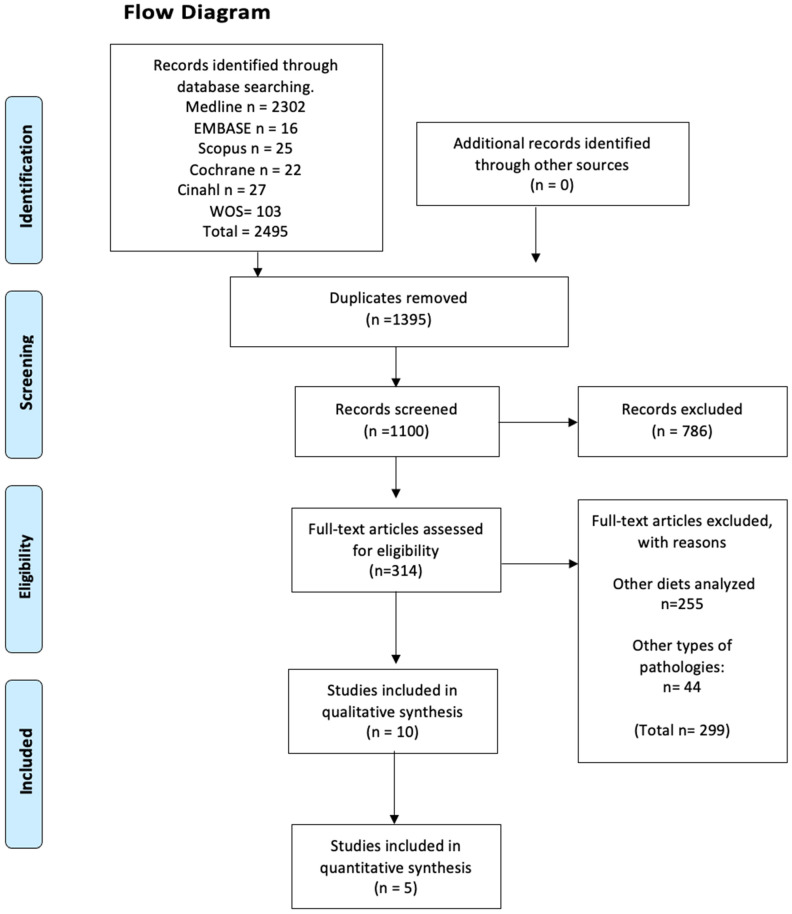
Flow chart and search results.

The electronic search retrieved a total of 2406 articles, of which 16 were potentially eligible for full-text review. The detailed steps of the article selection process for the systematic review are described in a flow diagram (Figure 1). Finally, the present review included a total of five randomized controlled trials that met the eligibility criteria [1,4,5,10,11]. The excluded studies and the reasons for their exclusion are available in Tables S1 and S2.

### 3.2. Study Characteristics

Table 1 summarizes the included studies. Of all included studies, only five eligible studies compared the DASH diet with other types of dietary treatment [1,4,5,10,11]. The studies’ publication dates were from 2011 to 2023. The overall population included 2013 patients (143 in the DASH diet group and 146 in the other diet modalities group). The mean ages were 65.7 (±4.7) and 67.2 (±3.8) years for the DASH diet and other diet modalities groups, respectively. The mean duration of follow-up was 8 weeks (range of 4 to 12).

### 3.3. Risk of Bias Assessment in Individual Studies

**Figure 2:**
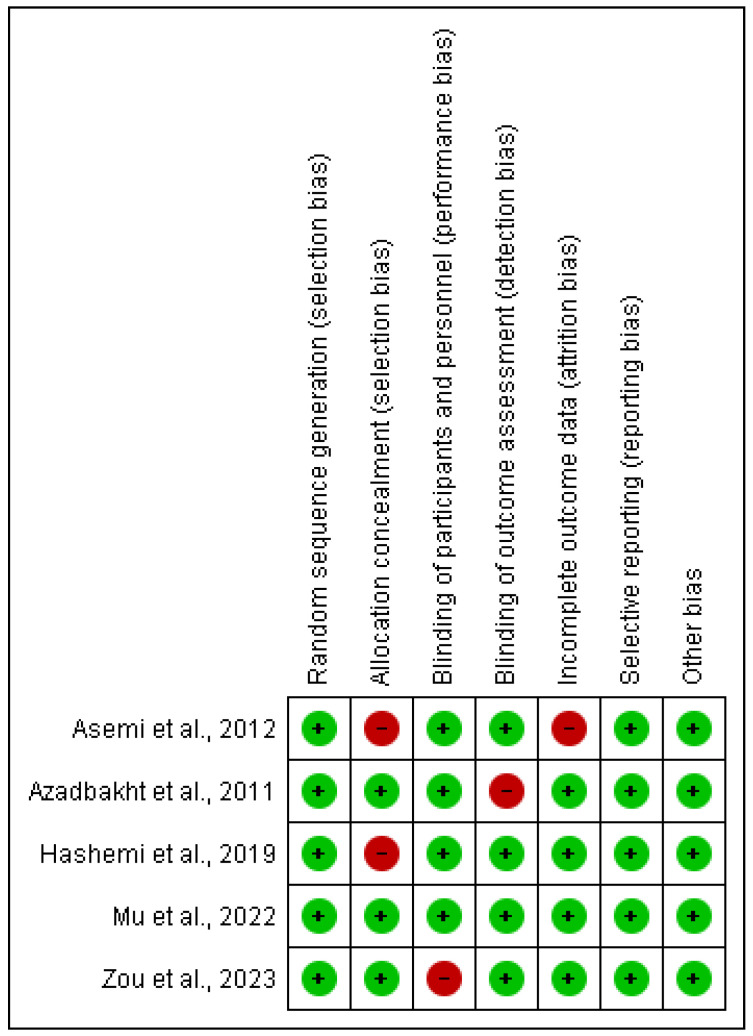
Risk of bias in included studies of meta-analyses [1,4,5,10,11].

The evaluation of RoB is presented in Figure 2. In the random sequence generation, 100% of the studies were classified as “low risk” [1,4,5,10,11]. In allocation concealment, 60% were classified as having a “low risk” of bias [4,10,11], while 40% presented a “high risk” [1,5]. For blinding of the participants and personnel, 80% of the trials were rated “low risk” of bias [1,4,5,11], while 20% received a “high risk” rating [10]. For blinding of the outcome assessment, 80% of the trials were rated “low risk” [1,4,5,10], and 20% as “high risk” [1]. For incomplete outcome data, 80% were rated “low risk” [1,4,10,11], and 20% as “high risk” [5]. Finally, for the selection of reported results, 100% of the trials were rated as “low risk” [1,4,5,10,11].

### 3.4. Descriptive Analysis of the Included Studies

Within this review, 10 studies could not be included in the meta-analysis because their outcomes and interventions could not be grouped with other studies. Of these investigations, five present statistically significant results, and their main findings are as follows: The DASH diet contributes to different degrees to the overall improvement of BP [13] in patients with and without metabolic syndrome. Along the same lines, a positive effect on SBP values is reported [2,6,13,15] as well as an improvement in DBP indices [2,3,13], with monitoring of ambulatory blood pressure during the day (ABPM Daytime Systolic; ABPM Daytime Diastolic; ABPM Nighttime Systolic) and for 24 h (ABPM 24-H Systolic; ABPM 24-H Diastolic) showing the DASH diet’s positive effect on these indices.

Associated with dyslipidemia and the effect of the DASH diet, a significant and positive contribution is reported for blood triglyceride values [15]. Regarding HDL, two different results are obtained: a decrease in these values [15] and an effective result for patients with and without metabolic syndrome [2].

Regarding the parameters associated with insulin resistance, greater reductions are observed in fasting values of insulin and the HOMA index through the application of the DASH diet, being statistically significant in patients with or without metabolic syndrome, without relevant differences between both groups [2]. Regarding HbA1c values, [6] reports a statistically significant contribution to this parameter’s improvement when using the DASH diet or a “very-low-carbohydrate diet” (VLC) in patients who had an HbA1c equal to or greater than 5.7%.

Finally, when considering a parameter related to the patients’ physical characteristics, [6] presents favorable results in the weight reduction of the individuals under study for both the VLC diet and the DASH diet.

### 3.5. Synthesis of Results

#### 3.5.1. SBP—1 to 4 Months’ Follow-Up

**Figure 3:**
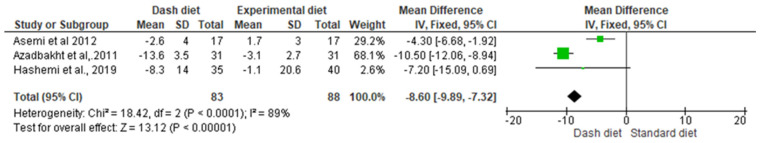
SBP—DASH diet vs. experimental diet, 1 to 4 months [1,5,11].

When the DASH diet was compared with an experimental diet, the three studies evaluated showed no significant differences (SMD = −8.60; CI = −9.89 to −7.32; *p* < 0.00001; Figure 3) [1,5,11]. The direction of the effect was consistent, and two of the CIs overlapped. Considerable statistical heterogeneity was observed (*I*^2^ = 89% and *p* < 0.0001). For this comparison, the funnel diagram graph shows an asymmetry, which indicates publication bias or factors that influence the results. The overall certainty of this evidence, based on the GRADE rating, was rated as low quality (Table S3).

#### 3.5.2. DBP—1 to 4 Months’ Follow-Up

**Figure 4:**
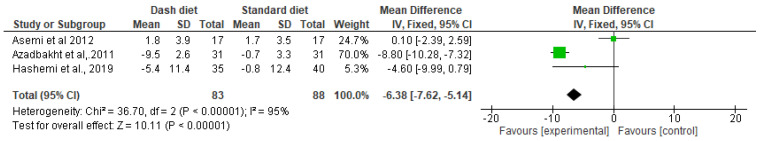
DBP—DASH diet vs. standard diet, 1 to 4 months [1,5,11].

When the DASH diet was compared with an experimental diet, the three studies evaluated showed no significant differences (SMD = −6.38; CI = −7.62 to −5.14; *p* < 0.00001; Figure 4) [1,5,11]. The direction of the effect was consistent, and two of the CIs overlapped. Considerable statistical heterogeneity was observed (*I*^2^ = 95% and *p* < 0.00001). For this comparison, the funnel diagram graph shows an asymmetry, which indicates publication bias or factors that influence the results. The overall certainty of this evidence, based on the GRADE rating, was rated as low quality (Table S3).

#### 3.5.3. HDL—1 to 2 Months’ Follow-Up

**Figure 5:**
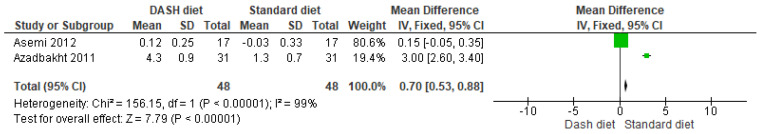
HDL—DASH diet vs. standard diet, 1 to 2 months [5,11].

When the DASH diet was compared with an experimental diet, the two studies evaluated showed no significant differences (SMD = 0.70; CI = 0.53 to 0.88; *p* < 0.00001; Figure 5) [5,11]. The direction of the effect was consistent, and two of the CIs overlapped. Considerable statistical heterogeneity was observed (*I*^2^ = 99% and *p* < 0.00001). For this comparison, the funnel diagram graph shows an asymmetry, which indicates publication bias or factors that influence the results. The overall certainty of this evidence, based on the GRADE rating, was rated as very low quality (Table S3).

#### 3.5.4. LDL—1 to 2 Months’ Follow-Up

**Figure 6:**
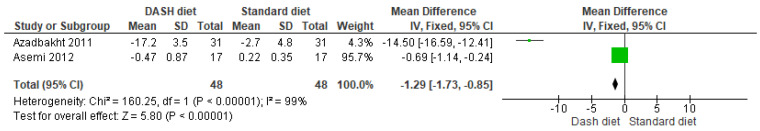
LDL—DASH diet vs. standard diet, 1 to 2 months [5,11].

When the DASH diet was compared with an experimental diet, the two studies evaluated showed no significant differences (SMD = −1.29; CI = −1.73 to −0.85; *p* < 0.00001; Figure 6) [5,11]. The direction of the effect was consistent, and two of the CIs overlapped. Considerable statistical heterogeneity was observed (*I*^2^ = 99% and *p* < 0.00001). For this comparison, the funnel diagram graph shows an asymmetry, which indicates publication bias or factors that influence the results. The overall certainty of this evidence, based on the GRADE rating, was rated as very low quality (Table S3).

#### 3.5.5. SBP SALT—8 Weeks’ Follow-Up

**Figure 7:**
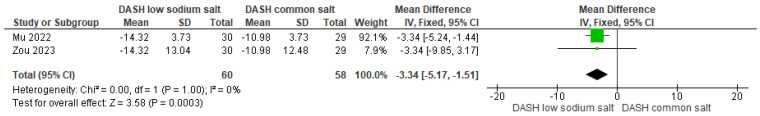
SBP—DASH low-sodium salt vs. DASH common salt, 8 weeks [4,10].

When the DASH diet was compared with another diet similar to DASH but with increased salt, the two studies evaluated showed no significant differences (SMD = −3.34; CI = −5.17 to −1.51; *p* < 0.0003; Figure 7) [4,10]. The direction of the effect was consistent, and two of the CIs overlapped. Low statistical heterogeneity was observed (*I*^2^ = 0% and *p* = 1.00). For this comparison, the funnel diagram graph shows an asymmetry, which indicates publication bias or factors that influence the results. The overall certainty of this evidence, based on the GRADE rating, was rated as low quality (Table S3).

#### 3.5.6. DBP SALT—8 Weeks’ Follow-Up

**Figure 8:**
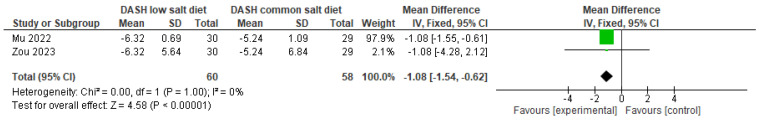
DBP—DASH low-sodium salt vs. DASH common salt, 8 weeks [4,10].

When the DASH diet was compared with an experimental diet, the two studies evaluated manifested no significant differences (SMD = −1.08; CI = −1.54 to −0.62; *p* < 0.00001; Figure 8) [4,10]. The direction of the effect was consistent, and two of the CIs overlapped. Considerable statistical heterogeneity was observed (*I*^2^ = 0% and *p* = 1.00). For this comparison, the funnel diagram graph shows an asymmetry, which indicates publication bias or factors that influence the results. The overall certainty of this evidence, based on the GRADE rating, was rated as low quality (Table S3).

## 4. Discussion

This systematic review and meta-analysis compared DASH diet modalities with other diet modalities for patients with any of the disorders considered in metabolic syndrome, such as those associated with blood pressure, glycemia, triglycerides, dyslipidemia, and abdominal obesity, with their different specifications and variants. The main findings were that, in the short and medium term, improvements were obtained in some of the parameters of these patients’ functional alteration, mainly in criteria such as systolic and diastolic blood pressure and HDL and LDL cholesterol. These same parameters showed statistically significant differences in favor of the DASH diet, except HDL, which was in favor of the other diets or the control group.

Previous reviews have reported the following: The 2021 review by Lari [18] showed as its main findings that the DASH diet helps reduce weight and improve systolic and diastolic blood pressure but did not show significant results in glycemic parameters, such as the HOMA test, and blood cholesterol parameters. However, it was descriptively reported that it could have beneficial effects in lowering or reducing blood lipids. In relation to this review, the present study carried out a more homogeneous comparison of parameters, such as blood pressure and blood cholesterol, giving exact groupings in sample, follow-up, and comparison parameters that were the most homogeneous between groups.

On the other hand, Juhasz [19] reported that the DASH diet should be preferred in treatment to reduce blood pressure problems in patients with polycystic ovaries. Our article differs because, in addition to evaluating the impact of the DASH diet on arterial hypertension, it evaluates other indicators, such as the effect on the different stages of dyslipidemia. Likewise, the patients of interest analyzed in this review included those who had any of the diagnostic characteristics for metabolic syndrome, with this being another distinction from what was reported by [19].

Finally, Salehi et al.’s 2013 review [20] showed as its main results an inverse relationship regarding the DASH diet, stating that by not using this diet, the increase in the development of cardiovascular conditions among the population not possessing alterations of this nature, or otherwise healthy, would be between 19% and 20%. For this reason, it is concluded that the DASH diet would act rather as a protective factor against possible unfavorable health outcomes. Considering this, if a comparison is made with our review, we instead carried out a search in relation to the treatment of the different constituent components of metabolic syndrome rather than its prevention.

Within the groupable parameters, DBP showed a statistically significant improvement in patients between 1 and 4 months with the DASH diet. Increased DBP is a clinical indicator in patients with metabolic syndrome, when presented theoretically and with high frequency. Along with this, associations have been established between increased diastolic blood pressure and the development of different cardiovascular risk factors. That being said, a decrease in this parameter through the implementation of the DASH diet demonstrates that its application is beneficial for patients’ health [21].

SBP is considered one of the diagnostic criteria for metabolic syndrome and, in general, is elevated in patients with this condition, thus implying an increase in the development of morbidity and mortality. In the effect on SBP between 1 and 4 months, a statistically significant difference was shown in favor of the DASH diet. The above and the well-known inverse relationship between SBP and the DASH diet demonstrates that its application together with lasting dietary changes leads to positive results for patients’ cardiovascular health, which is closely related to this diet’s reduced sodium consumption (among other factors), a micronutrient that is considered an important hypertensive agent when consumed in excess, given its characteristic as an active osmolyte, and that is largely responsible for high blood pressure disorder. Furthermore, we can add that if other types of factors are added to this, such as exercise, adequate management of mental status, and appropriate sleep habits, the effect could be greater [22].

On the other hand, regarding HDL, between 1 and 2 months, a statistically significant difference was obtained in favor of the standard diet; however, this result must be observed carefully, since it must be kept in mind that a healthy patient will have HDL parameters greater than those found in a patient with dyslipidemia, a condition which is one of the diagnostic criteria for metabolic syndrome and is closely related to HDL levels. For this reason, the use of the DASH diet in patients with metabolic syndrome is mostly beneficial for increasing this indicator compared to a standard diet, since HDL plays an important role by being responsible for transporting cholesterol located in the different regions of the body to the liver to be eliminated via the digestive tract. Furthermore, the importance of raising this parameter lies in reducing the probability of formation and development of atheromatous plaques on the walls of blood vessels, a disturbance that favors a slowing of blood flow and alteration in tissue perfusion, and which, in cases of poor prognosis, when fragmented can give rise to emboli, which will ultimately be responsible for causing cardiovascular, cerebrovascular, and pulmonary accidents, among others [23].

In the case of LDL between 1 and 2 months, a statistically significant difference could be seen in favor of the DASH diet. This parameter will directly influence one of the diagnostic criteria of metabolic syndrome, dyslipidemia, where its levels will frequently be elevated, which will imply negative health prognoses [12,14,16,17,24,25,26]. LDL is a low-density lipoprotein formed in the bloodstream, whose main function is to deliver cholesterol to different tissues. If its levels increase, it could cause cholesterol storage limits to be exceeded, which could be very harmful to the cardiovascular system, since, just as low levels of HDL can favor the development of atheroma plaques, they can also develop due to an increase in LDL, becoming an important risk factor for the appearance of coronary heart diseases. This is why, by observing that LDL levels are reduced by the use of the DASH diet in patients with metabolic syndrome, it can be seen that its application generates positive and beneficial results for this population [24].

Regarding the variations in systolic (SBP) and diastolic blood pressure (DBP), in DASH diets with consumption of different amounts of sodium for 8 weeks, it can be seen that there is a statistically significant difference in favor of the DASH diet, which implies a lower sodium consumption compared to the rest. As mentioned previously, sodium, having the characteristic of being an active osmolyte, will have significant repercussions on the body’s homeostasis, making special reference in this article to volume, and with it SBP and DBP. Given this, and according to the evidence, a directly proportional relationship between sodium levels (natremia, which is strongly influenced by dietary sodium intake) and blood pressure is mostly observed. For this reason, in patients with metabolic syndrome who have alterations in blood pressure, an adequate diet that involves low sodium consumption is essential, such as the version of the DASH diet with the lowest possible sodium consumption, to achieve adequate values and reduce the risk of developing various morbidities [25].

### Strengths and Limitations

We took the PRISMA statement as a guideline for the preparation of this systematic review and meta-analysis, and we also adhered to recommendations from the Cochrane collaboration manual and the synthesis of the quality of evidence evaluated with GRADE. Finally, to make our systematic review protocol transparent, we registered our reviews in PROSPERO, which means that our review used an appropriate and transparent methodology from an ethical point of view as well as reliable and verifiable information.

On the other hand, the limitations of this study are that, although we searched various data sources, there may have been articles that fit our inclusion criteria but were excluded from this review, given that they were found in the gray literature or were reported with terms or connectors different from those established in our search. Regarding the methodological limitations, we can address different factors such as the lack of an adequate sample size, unclear hidden allocation, lack of double-blind allocation, and variability of interventions used for the standard diet, which could have given rise to an overestimation of the effect size of interventions. Finally, due to the limited number of studies included in the meta-analysis, given the heterogeneity in the samples, interventions, and follow-ups, a limitation of our study could be that some relevant clinical parameters, such as the decrease in obesity or the decrease in blood glucose, could not be analyzed in relation to the effect of the DASH diet. Finally, our results should be interpreted with caution given the methodological limitations and heterogeneity of the included studies and the wide range of the strength of the available evidence.

## 5. Limitations

This review has limitations. First, the included studies may have publication bias: studies with different results that were found in literature not indexed in the selected databases may have been excluded. Second, there is a possibility that a more sensitive and specific search regarding the topic under study was not carried out. Lastly, the sample size of the articles was low, which makes this study present this limitation for data analysis.

## 6. Conclusions

MS patients present a variety of classic signs and symptoms that can alter their functionality and quality of life, in addition to having to consume a varied amount of drugs for a long time. In this review we found as a novelty the application of the DASH diet in the improvement of these patients through nutritional education, so we can infer that an improvement in the diet will always be beneficial for the health of the patients. But in this study, we were able to demonstrate that for patients with MS, some diets will have a greater effect on general health. In relation to the above, the DASH diet proved to be beneficial in parameters that patients with MS present as altered, and that these improvements can help considerably in the daily health of these patients; this is why we recommend that professionals who manage these pathologies promote the use of the DASH diet for the management of specific symptoms. In addition, we believe that new studies could always improve understanding and could better validate the use of the DASH diet or other diets in patients with MS.

## Figures and Tables

**Table 1:** Characteristics of the included studies.

Author	Country	Population		Intervention		Outcomes	Follow-Up	Results
		Sample Size (*n*)	Patient Mean Age (SD)	Intervention	Characteristics/Dose			
Paula et al., 2015 [3]	Brazil	CG: 20 EG: 20	CG: 62.5 (8.8) EG: 61.8 (8.1)	CG: American diet EG: DASH Diet	CG: 4 weeks. Total daily energy, 25 to 30 kcal of body weight, 50% to 60% of energy in the form of carbohydrates, 10% to 20% in the form of protein, and 25% to 30% in the form of total fat EG: 4 weeks with advice to increase walking. Total energy, 25 to 30 kcal of body weight, 55% of energy in the form of carbohydrates, 18% in the form of protein, and 27% in the form of total fat.	ABPM 24 h Systolic ABPM 24 h Dyastolic ABPM Daytime Systolic ABPM Daytime diastolic ABPM Nighttime systolic ABPM Nighttime diastolic	Not follow up	ABPM 24 h Systolic (*p* = 0.000) ABPM 24 h Dyastolic (*p* = 0.018) ABPM Daytime Systolic (*p* = 0.000) ABPM Daytime diastolic (*p* = 0.001) ABPM Nighttime systolic (*p* = 0.047) ABPM Nighttime diastolic (*p* = 0.129)
Mu et al., 2022 [10]	China	CG: 29 EG: 30	CG: 68.03 (3.82) EG: 70.00 (4.24)	CG: Modified DASH + Common salt EG: Modified DASH + Low-sodium salt	CG: 8 weeks. The amount of salt intake was controlled at 5 g/day per person. Common salt with sodium chloride content >99%. Special requirements for the modified DASH diet were the addition of brown rice, corn, soybeans, red beans, and other cereals to rice. EG: 8 weeks. The amount of salt intake was controlled at 5 g/day per person. Salt formula with 52% sodium. Special requirements for the modified DASH diet were the addition of brown rice, corn, soybeans, red beans, and other cereals to rice; eating lean meats, poultry, fish, and other meats < 120 g/day, with less red meat and more white meat.	SBP DBP Sodium (mmol/24 h) Potassium (mmol/24 h) Sodium/Potassium ratio Chlorine (mmol/24 h) Calcium (mmol/24 h) Magnesium (mmol/24 h) Phosphorus (mmol/24 h) Albumin (μmol/L) Creatinine (mmol/L) UACR (mg/mmol)	Not follow up	SBP (*p* = 0.929) DBP (*p* = 0.093) Sodium (mmol/24 h) (*p* = 0.027) Potassium (mmol/24 h) (*p* = 0.306) Sodium/Potassium ratio (*p* = 0.227) Chlorine (mmol/24 h) (*p* = 0.035) Calcium (mmol/24 h) (*p* = 0.612) Magnesium (mmol/24 h) (*p* = 0.236) Phosphorus (mmol/24 h) (*p* = 0.269) Albumin (μmol/L) (*p* = 0.053) Creatinine (mmol/L) (*p* = 0.013) UACR (mg/mmol) (*p* = 0.005)
Saslow et al., 2023 [6]	USA	CG: 23 CG + Support: 22 EG: 25 EG + Support: 24	VLC: 60.09 (6.03) VLC + Support: 55.18 (10.48) DASH: 58.40 (8.11) DASH + Support: 60.21 (6.19)	CG: Very low carbohydrate diet. CG + Support: Very low carbohydrate diet + support with information. EG: DASH diet. EG + Support: DASH diet + support with information.	VLC: 4 months Decrease carbohydrate intake to 20 to 35 g of non-fiber carbohydrates per day. VLC + Support: 4 months Decrease carbohydrate intake to 20 to 35 g of non-fiber carbohydrates per day. DASH: 4 months Limit sodium to <2300 mg daily and fat intake to 20% to 30% of calories per day. DASH + Support: 4 months Limit sodium to < 2300 mg daily and fat intake to 20% to 30% of calories per day. Recommended consumption of a variety of fruits and vegetables, lean meats and fish, whole grains, and low-fat dairy products.	SBP HbA1c (%) Weight (lb)	No follow-up	* SBP (*p* = 0.046) HbA1c (%) (*p* = 0.034) Weight (lb) (*p* = 0.0003) ** SBP (*p* = 0.49) HbA1c (%) (*p* = 0.51) Weight (lb) (*p* = 0.18)
Belfort et al., 2023 [12]	Brazil	CG: 35 EG: 3	No report	CG: Standard diet. EG: Adapted DASH diet.	CG: 18 weeks Oatmeal ration (250 gr), skimmed milk (1–2%, 300 gr), and extra virgin olive oil 500 mL were provided. EG: 18 weeks Provides a ration of seeds (200 gr), nuts (150 gr), skimmed milk (280 gr), and 500 mL of extra virgin olive oil.	SBP (mmHg) DBP (mmHg) LDL-cholesterol (mg/dL) HDL-cholesterol (mg/dL) Glycated hemoglobin (%) Triglycerides (mg/dL) Glutathione peroxidase (µmol/L) CRP (mg/dL) Total cholesterol (mg/dL)	No reported	SBP (*p* = 0.65) DBP (*p* = 0.36) LDL-cholesterol (*p* = 0.43) HDL-cholesterol (*p* = 0.71) Glycated hemoglobin (*p* = 0.12) Triglycerides (*p* = 0.4) Glutathione peroxidase (*p* = 0.09) CRP (*p* = 0.09) Total cholesterol (*p* = 0.48)
Zou et al., 2023 [4]	China	CG: 29 EG: 30	CG: 68.03 (3.82) EG: 70.00 (4.24)	CG: Common salt EG: Low- sodium salt	CG: 8 weeks standard diet EG: 8 weeks Rice: whole grain. Mainly white meat (poultry or fish). Low-fat milk. Sufficient vegetables (mainly green leafy vegetables).	SBP (mmHg) DBP (mmHg) FBG (mmol/L) PBG (mmol/L) BMI (kg/cm^2^) WHR (cm)	Not follow up	SBP (*p* = 0.231) DBP (*p* = 0.698) FBG (*p* = 0.77) PBG (*p* = 0.002) BMI (*p* = 0.832) WHR (*p* = 0.914)
Hashemi et al., 2019 [1]	Iran	CG: 40 EG: 35	CG: <50 EG: <50	CG: Control group diet EG: DASH diet	CG: 12 weeks 18% protein, 52% carbohydrates, 30% fat. EG: 12 weeks 8 to 10 daily servings of fruits and vegetables; 4 to 5 weekly servings of nuts, seeds or beans.	SBP (mmHg) DBP (mmHg)	No follow-up	SBP (*p* = 0.813) DBP (*p* = 0.921)
Hikmat et al., 2013 [13]	USA	CG: 133 EG: 138 AG: 140	CG: 45.4 (11.4) EG: 44.5 (10.5) AG: 48.4 (11)	CG: Protein, Carbohydrates, Fat Diet EG: DASH Diet	CG: Protein, carbohydrate, and fat diet. EG: DASH diet: 12 weeks. 8 to 10 daily servings of fruits and vegetables; 4 to 5 weekly servings of nuts, seeds, or beans. At least six daily servings from a grain group (half of them should be whole grains).	BMI BP TG HDL SBP DBP	No follow-up	BMI (*p* = 0.001) BP (*p* = 0.001) TG (*p* = 0.001) HDL (*p* = 0.001) SBP (*p* = 0.001) DBP (*p* = 0.11)
Azadbakht et al., 2011 [11]	Iran	CG: 31 EG:31	CG:Not reported EG:Not reported	CG: Protein, carbohydrate and fat EG: Dieta DASH	CG: Control diet: 8 weeks 50 to 60% carbohydrates; 15 to 20% protein; 30% total fat; 5% of caloric intake comes from simple sugars. EG: DASH diet: 8 weeks Rich in fruits, vegetables, whole grains, and low-fat dairy products and low in saturated fat, total fat, cholesterol, refined grains, and sweets. Amount of sodium consumed was 2400 mg per day	SBP (mmHg) DBP (mmHg) FBG (mg/dL) A1C (%) TG (mg/dL) HDL-C (mg/dL) LDL-C (mg/dL)	No follow-up	SBP (*p* = 0.39) DBP (*p* = 0.95) FBG (*p* = 0.73) A1C (*p* = 0.19) TG (*p* = 0.17) HDL-C (*p* = 0.97) LDL-C (*p* = 0.23)
Shenoy et al., 2010 [14]	USA	CG: 27 EG: 27 AG: 27	CG: 50.1 (5.1) EG: 51.2 (7.4) AG: 48.0 (7.7)	CG: not reported EG: DASH Diet and Vegetable Juice 8 oz AG: DASH Diet and Vegetable Juice 16 oz	CG: not reported EG: DASH diet without vegetable juice: 12 weeks. Men 1800 Kcal diet, and women 1600 Kcal. EG: DASH diet of 8 fluid ounces of low-sodium vegetable juice per day: 12 weeks. Men 1800 Kcal diet, and women 1600 Kcal. EG: DASH diet of 16 fluid ounces of low-sodium vegetable juice per day: 12 weeks. Men 1800 kcal diet and women 1600 kcal diet.	HDL: (mg/dL; %) Blood Glucose: (mg/dL; %) BMI: (KG) SBP: (mmHg) DBP: (mmHg) TG: (mg/dL; %)	No follow-up	HDL: Blood Glucose: BMI: SBP: DBP: TG:
Lien et al., 2007 [2]	USA	CG: 397 EG: 399	CG: 49.9 (9.0) EG:49.7 (8.6)	CG: DASH diet with metabolic syndrome EG: DASH diet	CG: Not reported Group receiving the intervention plus the DASH dietary pattern. EG: Group receiving an intensive behavioral intervention based on established lifestyle modifications to lower blood pressure and receiving the intervention plus the DASH dietary pattern.	TG (mg/dL) SBP (mmHg) DBP (mmHg) LDL-C (mg/dL) HDL-C (mg/dL) FG (mmol/L) FI (micro-IU/mL) HOMA	No follow-up	TG: (*p* = 0.0001) SBP: (*p* = 0.0003) DBP: (*p* = 0.0001) LDL-C: (*p* = 0.3157) HDL-C: (*p* = 0.0001) FG: (*p* = 0.0001) FI: (*p* = 0.0001) HOMA: (*p* = 0.0001)
Asemi et al., 2012 [5]	Iran	CG:17 EG:17	CG: 29.4 (6.2) EG: 30.7 (6.7)	CG: Standard Diet EG: DASH Diet	CG: No reported EG: No reported	BMI (kg/m^2^) HDL (mmol/L) LDL (mmol/L) SBP (mmHg) DBP (mmHg) GTT (mmol/L)	No follow-up	BMI: (*p* = 0.15) HDL: (*p* = 0.17) LDL: (*p* = 0.005) SBP:(*p* = 0.001) DBP: (*p* = 0.98) GTT: (*p* = 0.001)
Root et al., 2013 [15]	USA	CG: Not reported EG:Not reported AG:Not reported AG:Not reported	CG:Not reported EG: 30≥ AG: 30≥ AG: 30≥	CG: Not reported EG: High-carbohydrate DASH-like diet AG: High-protein DASH-like diet AG: High-unsaturated fat DASH-like diet	CG: Not reported EG: High-carbohydrate DASH-like diet 6 weeks. Diet based on 58% carbohydrates, 15% protein, and 27% fat. 5.5% plant-based protein, 13% monounsaturated fats, and 8% polyunsaturated fats.	Glucose (mmol/L) HDL-C (mmol/L) TG (mmol/L) SBP (mmHg)	5 years	Glucose: EG: (*p* = 0.99) AG: (*p* = 0.78) AG: (*p* = 0.34) HDL-C: EG: (*p* = 0.014) AG: (*p* ≤ 0.001) AG: (*p* = 0.47) TG: EG: (*p* = 0.57) AG: (*p* ≤ 0.001) AG: (*p* = 0.001) SBP: EG: (*p* ≤ 0.001) AG: (*p* ≤ 0.001) AG: (*p* ≤ 0.001)
Al-Solaiman et al., 2010 [16]	USA	CG: 30 EG: 30	CG: Not reported. EG: Not reported.	CG: ULFV-S EG: DASH	CG: Diet with an average of one fruit and one vegetable (ULFV), ∼1700 mg potassium, 250 mg magnesium, and 11 g of fiber daily; supplemented with potassium, magnesium, and fiber (ULFV-S) to match DASH, or DASH itself. EG: ∼50% carbohydrate, 35% fat, and 15% protein with 3000 mg sodium and 700 mg calcium daily.	Not reported	No follow-up	Not reported
Blumental et al., 2010 [17]	USA	CG: 49 EG: 46 AG: 49	CG: 52.0 (10) EG: 51.8 (10) AG: 52.3 (10)	CG: UC EG: DASH-A AG: DASH-WM	CG: Regular diet: 4 weeks with the cases, 2 weeks with controls; the following weeks, only an indication to follow the diet was given: isocaloric diet to maintain weight proteins 16.5%, saturated fat 36.8%, fiber 16 G EG: DASH diet alone 4 weeks with the cases, 2 weeks with controls; the following weeks, only an indication to follow the diet was given: isocaloric diet to maintain weight proteins 19.4%, saturated fat 27.8%, fiber 26%	FG (mg/dL) FI (μU/mL) Glucosa AUC (mg/dl·minutes) TG (mg/dL) Total Cholesterol (mg/dL) Low-Density Lipoprotein-Cholesterol (mg/dL) High-Density Lipoprotein-Cholesterol (mg/dL)	4 months	FG: GC: (*p*=) EG: (*p*=) AG: (*p*=) FI: GC: (*p*=) EG: (*p*=) AG: (*p*=) Glucosa AUC: GC: (*p*=) EG: (*p*=) AG: (*p*=) TG: GC: (*p*=) EG: (*p*=) AG: (*p*=) Total Cholesterol: GC: (*p*=) EG: (*p*=) AG: (*p*=) Low-Density Lipoprotein-Cholesterol: GC: (*p*=) EG: (*p*=) AG: (*p*=) High-Density Lipoprotein-Cholesterol: GC: (*p*=) EG: (*p*=) AG: (*p*=)

SD: Standard deviation; CG: Control group; EG: Experimental group; SBP: Systolic blood pressure; DBP: Diastolic blood pressure; VLC: Very Low Carbohydrate; HDL-C: HDL cholesterol; LDL-C: LDL cholesterol; FBG: fasting blood glucose; TG: triglyceride; FG: fasting glucose; FI: fasting insulin; SS: Salt sensitive; SR: Salt resistant. * Between VLC and DASH diet; ** Between groups with and without support.

## Supplementary Materials

The following supporting information can be downloaded at: https://www.mdpi.com/article/10.3390/nu16183054/s1, Table S1: Search strategy for each database analyzed, with the result of papers found; Table S2: Excluded studies and the reasons for their exclusion; Table S3: Grade comparison includes.

## References

[1] Hashemi R., Rahimlou M., Baghdadian S., Manafi M. (2019). Investigating the effect of DASH diet on blood pressure of patients with type 2 diabetes and prehypertension: Randomized clinical trial. Diabetes Metab. Syndr..

[2] Lien L.F., Brown A.J., Ard J.D., Loria C., Erlinger T.P., Feldstein A.C., Lin P.-H., Champagne C.M., King A.C., McGuire H.L. (2007). Effects of PREMIER lifestyle modifications on participants with and without the metabolic syndrome. Hypertension.

[3] Paula T.P., Viana L.V., Neto A.T.Z., Leitão C.B., Gross J.L., Azevedo M.J. (2015). Effects of the DASH diet and walking on blood pressure in patients with type 2 diabetes and uncontrolled hypertension: A randomized controlled trial. J. Clin. Hypertens..

[4] Mu L., Zou Y., Tang J., Zhang F., Chen D., Mu L., Xu H., Yu P., Ren Y., Mei Y. (2023). Effect of low-sodium salt applied to Chinese modified DASH diet on arterial stiffness in older patients with hypertension and type 2 diabetes. Nutr. Hosp..

[5] Asemi Z., Tabassi Z., Samimi M., Fahiminejad T., Esmaillzadeh A. (2013). Favourable effects of the Dietary Approaches to Stop Hypertension diet on glucose tolerance and lipid profiles in gestational diabetes: A randomised clinical trial. Br. J. Nutr..

[6] Saslow L.R., Jones L.M., Sen A., Wolfson J.A., Diez H.L., O’brien A., Leung C.W., Bayandorian H., Daubenmier J., Missel A.L. (2023). Comparing very low-carbohydrate vs DASH diets for overweight or obese adults with hypertension and prediabetes or type 2 diabetes: A randomized trial. Ann. Fam. Med..

[7] Page M.J., McKenzie J.E., Bossuyt P.M., Boutron I., Hoffmann T.C., Mulrow C.D., Shamseer L., Tetzlaff J.M., Akl E.A., Brennan S.E. (2021). The PRISMA 2020 statement: An updated guideline for reporting systematic reviews. BMJ.

[8] Eldridge S., Campbell M.K., Campbell M.J., Revised Cochrane Risk of Bias Tool for Randomized Trials (RoB2) AdditionalConsiderations for Cluster-Randomized Trials (RoB 2 CRT). https://methods.cochrane.org/bias/resources/rob-2-revised-cochrane-risk-bias-tool-randomized-trials.

[9] Fritz C.O., Morris P.E., Richler J.J. (2012). Effect size estimates: Current use, calculations, and interpretation. J. Exp. Psychol. Gen..

[10] Mu L., Mu L., Mu L., Mu L., Yu P., Yu P., Xu H., Xu H., Gong T., Gong T. (2022). Efecto de la reducción de sodio basada en la dieta DASH sobre la presión arterial en pacientes hipertensos con diabetes tipo 2. Nutr. Hosp..

[11] Azadbakht L., Fard N.R.P., Karimi M., Baghaei M.H., Surkan P.J., Rahimi M., Esmaillzadeh A., Willett W.C. (2011). Effects of the Dietary Approaches to Stop Hypertension (DASH) eating plan on cardiovascular risks Among type 2 diabetic patients. Diabetes Care.

[12] Belfort G.P., Padilha P.d.C., Farias D.R., da Silva L.B.G., dos Santos K., Gomes E.d.S., Lima T.S.V., Bornia R.B.R.G., Rezende K.B.C., Saunders C. (2023). Effect of the Dietary Approaches to Stop Hypertension (DASH) diet on the development of preeclampsia and metabolic outcomes in pregnant women with pre-existing diabetes mellitus: A randomised, controlled, single-blind trial. J. Nutr. Sci..

[13] Hikmat F., Appel L.J. (2014). Effects of the DASH diet on blood pressure in patients with and without metabolic syndrome: Results from the DASH trial. J. Hum. Hypertens..

[14] Shenoy S.F., Poston W.S., Reeves R.S., Kazaks A.G., Holt R.R., Keen C.L., Chen H.J., Haddock C.K., Winters B.L., Khoo C.S.H. (2010). Weight loss in individuals with metabolic syndrome given DASH diet counseling when provided a low sodium vegetable juice: A randomized controlled trial. Nutr. J..

[15] Root M.M., Dawson H.R. (2013). DASH-like diets high in protein or monounsaturated fats improve metabolic syndrome and calculated vascular risk. Int. J. Vitam. Nutr. Res..

[16] Al-Solaiman Y., Jesri A., Mountford W.K., Lackland D.T., Zhao Y., Egan B.M. (2010). DASH lowers blood pressure in obese hypertensives beyond potassium, magnesium and fibre. J. Hum. Hypertens..

[17] Blumenthal J.A., Babyak M.A., Sherwood A., Craighead L., Lin P.-H., Johnson J., Watkins L.L., Wang J.T., Kuhn C., Feinglos M. (2010). Effects of the Dietary Approaches to Stop Hypertension diet alone and in combination with exercise and caloric restriction on insulin sensitivity and lipids. Hypertension.

[18] Lari A., Sohouli M.H., Fatahi S., Cerqueira H.S., Santos H.O., Pourrajab B., Rezaei M., Saneie S., Rahideh S.T. (2021). The effects of the Dietary Approaches to Stop Hypertension (DASH) diet on metabolic risk factors in patients with chronic disease: A systematic review and meta-analysis of randomized controlled trials. Nutr. Metab. Cardiovasc. Dis..

[19] Juhász A.E., Stubnya M.P., Teutsch B., Gede N., Hegyi P., Nyirády P., Bánhidy F., Ács N., Juhász R. (2024). Clasificación de las intervenciones dietéticas según su eficacia en el tratamiento del síndrome de ovario poliquístico: Una revisión sistemática y un metanálisis en red. Reprod. Health.

[20] Salehi-Abargouei A., Maghsoudi Z., Shirani F., Azadbakht L. (2013). Effects of Dietary Approaches to Stop Hypertension (DASH)-style diet on fatal or nonfatal cardiovascular diseases—Incidence: A systematic review and meta-analysis on observational prospective studies. Nutrition.

[21] Zidek W., Naditch-Brûlé L., Perlini S., Farsang C., E Kjeldsen S. (2009). Control de la presión arterial y componentes del síndrome metabólico: La encuesta GOOD. Cardiovasc. Diabetol..

[22] Sacks F.M., Svetkey L.P., Vollmer W.M., Appel L.J., Bray G.A., Harsha D., Obarzanek E., Conlin P.R., Miller E.R., Simons-Morton D.G. (2001). Effects on blood pressure of reduced dietary sodium and the dietary approaches to stop hypertension (DASH) diet. N. Engl. J. Med..

[23] Said M.S., El Sayed I.T., Ibrahim E.E., Khafagy G.M. (2021). Effect of DASH diet versus healthy dietary advice on the estimated atherosclerotic cardiovascular disease risk. J. Prim. Care Community Health.

[24] Santos A.R. Caso Clínico: Efeitos da Dieta Dietary Approaches to Stop Hypertension (Dash) No Controle Metabólico. https://expertisecia.com/wp-content/uploads/simple-file-list/TCC-ADRIANA-REIS-Final-1_1.pdf.

[25] Juraschek S.P., Miller E.R., Weaver C.M., Appel L.J. (2017). Effects of sodium reduction and the DASH diet in relation to baseline blood pressure. J. Am. Coll. Cardiol..

[26] Al-Solaiman Y., Jesri A., Zhao Y., Morrow J.D., Egan B.M. (2009). Low-sodium DASH reduces oxidative stress and improves vascular function in salt-sensitive humans. J. Hum..

