# Supplementary material for: Effectiveness of DASH Diet versus Other Diet Modalities in Patients with Metabolic Syndrome: A Systematic Review and Meta-Analysis
Source: Nutrients. 2024 Sep 10;16(18):3054. doi: 10.3390/nu16183054 (PMC11434995; doi:10.3390/nu16183054)
Supplement: Supplementary file 1 [file nutrients-16-03054-s001.zip › nutrients-3165628-supplementary.pdf]

Supplementary Tables

Table S1: Search strategy for each database analyzed, with the result of papers found

Database	Search strategy	Results	
		**12-05-24**	**01-08-24**
**Medline**	(((("dietary approaches to stop hypertension"[MeSH Terms] OR ("dietary"[All Fields] AND "approaches"[All Fields] AND "stop"[All Fields] AND "hypertension"[All Fields]) OR "dietary approaches to stop hypertension"[All Fields] OR ("Dash"[All Fields] AND "diet"[All Fields]) OR "dash diet"[All Fields] OR ("Dash"[All Fields] AND ("food"[MeSH Terms] OR "food"[All Fields])) OR ("Dash"[All Fields] AND ("diet"[MeSH Terms] OR "diet"[All Fields] OR "dietary"[All Fields] OR "dietaries"[All Fields]))) AND ("metabolic syndrome"[MeSH Terms] OR ("metabolic"[All Fields] AND "syndrome"[All Fields]) OR "metabolic syndrome"[All Fields])) OR ("x"[All Fields] AND ("syndrom"[All Fields] OR "syndromal"[All Fields] OR "syndromally"[All Fields] OR "syndrome"[MeSH Terms] OR "syndrome"[All Fields] OR "syndromes"[All Fields] OR "syndrome s"[All Fields] OR "syndromic"[All Fields] OR "syndroms"[All Fields]))) NOT ("animals"[MeSH Terms:noexp] OR "animals"[All Fields])) AND ((clinicaltrial[Filter] OR randomizedcontrolledtrial[Filter]) AND (humans[Filter]))	2301	2302
**EMBASE**	Dash diet OR Dash food OR Dash dietary AND metabolic syndrome OR x syndrome NOT animals	15	16
**SCOPUS**	Dash diet OR Dash food OR Dash dietary AND metabolic syndrome OR x syndrome NOT animals	24	25

**Cochrane**	Dash diet OR Dash food OR Dash dietary AND metabolic syndrome OR x syndrome NOT animals	22	22
**Cinahl**	Dash diet OR Dash food OR Dash dietary AND metabolic syndrome OR x syndrome NOT animals	26	28
**WOS**	Dash diet OR Dash food OR Dash dietary AND metabolic syndrome OR x syndrome NOT animals	13	13
**All**	Total	2401	2406

* All searches were carried out on August 1, 2024.

Table S2: Excluded studies and the reasons for their exclusion.

N°	Reference	Reason
1	Paula TP, Viana LV, Neto AT, Leitão CB, Gross JL, Azevedo MJ. Effects of the DASH Diet and Walking on Blood Pressure in Patients With Type 2 Diabetes and Uncontrolled Hypertension: A Randomized Controlled Trial. J Clin Hypertens (Greenwich). 2015 Nov;17(11):895-901. doi: 10.1111/jch.12597. Epub 2015 Jun 4. PMID: 26041459; PMCID: PMC8031764.	The patients only presented hypertension as a baseline condition and no metabolic syndrome.
2	Sacks FM, Svetkey LP, Vollmer WM, Appel LJ, Bray GA, Harsha D, Obarzanek E, Conlin PR, Miller ER 3rd, Simons-Morton DG, Karanja N, Lin PH; DASH-Sodium Collaborative Research Group. Effects on blood pressure of reduced dietary sodium and the Dietary Approaches to Stop Hypertension (DASH) diet. DASH-Sodium Collaborative Research Group. N Engl J Med. 2001 Jan 4;344(1):3-10. doi: 10.1056/NEJM200101043440101. PMID: 11136953.	The patients only presented hypertension as a baseline condition and no metabolic syndrome.
3	Juraschek SP, Miller ER 3rd, Weaver CM, Appel LJ. Effects of Sodium Reduction and the DASH Diet in Relation to Baseline Blood Pressure. J Am Coll Cardiol. 2017 Dec 12;70(23):2841-2848. doi: 10.1016/j.jacc.2017.10.011. Epub 2017 Nov 12. PMID: 29141784; PMCID: PMC5742671.	The patients only presented hypertension as a baseline condition and no metabolic syndrome.
4	Filippou C, Thomopoulos C, Konstantinidis D, Siafi E, Tatakis F, Manta E, Drogkaris S, Polyzos D, Kyriazopoulos K, Grigoriou K, Tousoulis D, Tsioufis K. DASH vs. Mediterranean diet on a salt restriction background in adults with high normal blood pressure or grade 1 hypertension: A randomized controlled trial. Clin Nutr. 2023 Oct;42(10):1807-1816. doi: 10.1016/j.clnu.2023.08.011. Epub 2023 Aug 18. PMID: 37625311.	The patients only presented hypertension as a baseline condition and no metabolic

		syndrome.
5	Kucharska A, Gajewska D, Kiedrowski M, Sińska B, Juszczyk G, Czerw A, Augustynowicz A, Bobiński K, Deptała A, Niegowska J. The impact of individualised nutritional therapy according to DASH diet on blood pressure, body mass, and selected biochemical parameters in overweight/obese patients with primary arterial hypertension: a prospective randomised study. Kardiol Pol. 2018;76(1):158-165. doi: 10.5603/KP.a2017.0184. Epub 2017 Oct 5. PMID: 28980293.	The patients only presented hypertension as a baseline condition and no metabolic syndrome.
6	Wright KD, Klatt MD, Adams IR, Nguyen CM, Mion LC, Tan A, Monroe TB, Rose KM, Scharre DW. Mindfulness in Motion and Dietary Approaches to Stop Hypertension (DASH) in Hypertensive African Americans. J Am Geriatr Soc. 2021 Mar;69(3):773-778. doi: 10.1111/jgs.16947. Epub 2020 Nov 23. PMID: 33227157; PMCID: PMC8329944.	The patients only presented hypertension as a baseline condition and no metabolic syndrome.
7	Castilla-Ojo N, Turkson-Ocran RA, Conlin PR, Appel LJ, Miller ER 3rd, Juraschek SP. Effects of the DASH diet and losartan on serum urate among adults with hypertension: Results of a randomized trial. J Clin Hypertens (Greenwich). 2023 Oct;25(10):915-922. doi: 10.1111/jch.14721. Epub 2023 Sep 11. PMID: 37695134; PMCID: PMC10560966.	The patients only presented hypertension as a baseline condition and no metabolic syndrome.
8	Belanger MJ, Kovell LC, Turkson-Ocran RA, Mukamal KJ, Liu X, Appel LJ, Miller ER 3rd, Sacks FM, Christenson RH, Rebuck H, Chang AR, Juraschek SP. Effects of the Dietary Approaches to Stop Hypertension Diet on Change in Cardiac Biomarkers Over Time: Results From the DASH-Sodium Trial. J Am Heart Assoc. 2023 Jan 17;12(2):e026684. doi: 10.1161/JAHA.122.026684. Epub 2023 Jan 11. PMID: 36628985; PMCID: PMC9939071.	The patients only presented hypertension as a baseline condition and no metabolic syndrome.
9	Couch SC, Saelens BE, Khoury PR, Dart KB, Hinn K, Mitsnefes MM, Daniels SR, Urbina EM. Dietary Approaches to Stop Hypertension Dietary Intervention Improves Blood Pressure and Vascular Health in Youth With Elevated Blood Pressure. Hypertension. 2021 Jan;77(1):241-251. doi: 10.1161/HYPERTENSIONAHA.120.16156. Epub 2020 Nov 16. PMID: 33190559; PMCID: PMC7725858.	The patients only presented hypertension as a baseline condition and no metabolic syndrome.
10	Pourafshar S, Nicchitta M, Tyson CC, Svetkey LP, Corcoran DL, Bain JR, Muehlbauer MJ, Ilkayeva O, O'Connell TM, Lin PH, Scialla JJ. Urine and Plasma Metabolome of Healthy Adults Consuming the DASH (Dietary Approaches to Stop Hypertension) Diet: A Randomized Pilot Feeding Study. Nutrients. 2021 May 22;13(6):1768. doi: 10.3390/nu13061768. PMID: 34067295; PMCID: PMC8224662.	The patients only presented hypertension as a baseline condition and no metabolic syndrome.
11	Saslow LR, Jones LM, Sen A, Wolfson JA, Diez HL, O'Brien A, Leung CW, Bayandorian H, Daubenmier J, Missel AL, Richardson C. Comparing Very	The patients only presented

	Low-Carbohydrate vs DASH Diets for Overweight or Obese Adults With Hypertension and Prediabetes or Type 2 Diabetes: A Randomized Trial. Ann Fam Med. 2023 May-Jun;21(3):256-263. doi:	prediabetes or type 2 diabetes as a baseline condition and no metabolic syndrome.
12	Sun B, Williams JS, Svetkey LP, Kolatkar NS, Conlin PR. Beta2-adrenergic receptor genotype affects the renin-angiotensin-aldosterone system response to the Dietary Approaches to Stop Hypertension (DASH) dietary pattern. Am J Clin Nutr. 2010 Aug;92(2):444-9. doi: 10.3945/ajcn.2009.28924. Epub 2010 Jun 2. PMID: 20519561; PMCID: PMC2904038.	The patients only presented hypertension as a baseline condition and no metabolic syndrome.
13	Santos KD, Rosado EL, da Fonseca ACP, Belfort GP, da Silva LBG, Ribeiro-Alves M, Zembrzuski VM, Martínez JA, Saunders C. FTO and ADRB2 Genetic Polymorphisms Are Risk Factors for Earlier Excessive Gestational Weight Gain in Pregnant Women with Pregestational Diabetes Mellitus: Results of a Randomized Nutrigenetic Trial. Nutrients. 2022 Mar 1;14(5):1050. doi: 10.3390/nu14051050. PMID: 35268025; PMCID: PMC8912276.	The patients only presented type 2 diabetes as a baseline condition and no metabolic syndrome.
14	Tilves C, Yeh HC, Maruthur N, Juraschek SP, Miller ER, Appel LJ, Mueller NT. A behavioral weight-loss intervention, but not metformin, decreases a marker of gut barrier permeability: results from the SPIRIT randomized trial. Int J Obes (Lond). 2022 Mar;46(3):655-660. doi: 10.1038/s41366-021-01039-2. Epub 2022 Jan 6. PMID: 34987204; PMCID: PMC8881332.	Evaluates safety in patients with cardiovascular diseases and diabetes.
15	O'Connor LE, Li J, Sayer RD, Hennessy JE, Campbell WW. Short-Term Effects of Healthy Eating Pattern Cycling on Cardiovascular Disease Risk Factors: Pooled Results from Two Randomized Controlled Trials. Nutrients. 2018 Nov 10;10(11):1725. doi: 10.3390/nu10111725. PMID: 30423846; PMCID: PMC6266045.	The patients only presented cardiovascular disease as a baseline condition and no metabolic syndrome.

Table S3: Grade comparison includes.

Certainty assessment							№ of patients		Effect		Quality of evidence (GRADE)	Importance
№ of studies	Study design	Risk of bias	Inconsistency	Indirectness	Imprecision	Other considerations	Duloxetine	Control group	Relative (95% CI)	SMD (95% CI)		

SBP one to four months follow up.

3	RCT	Not Serious	Serious	Not serious	Serious	None	83	88	-	-8.60 (-9.89 to -7.32)	⊕⊕◯◯ Low	CRITICAL

DBP one to four months follow up.

3	RCT	Not Serious	Very serious	Not serious	Serious	None	83	88	-	-6.38 (-7.62 to -5.14)	⊕⊕◯◯ Low	CRITICAL

HDL one to two months follow up.

2	RCT	Not serious	Very serious	Not serious	Serious	None	48	48	-	0.70 (-0.53 to 0.88)	⊕⊕◯◯ Low	CRITICAL

LDL one to two months follow up.

2	RCT	Not Serious	Very serious	Not serious	Serious	None	48	48	-	-1.29 (-0.73 to -0.85)	⊕⊕◯◯ Low	IMPORTANT

SBP SALT eight weeks follow up.

2	RCT	Not serious	Very serious	Not serious	Serious	None	60	58	-	-3.34 (-5.17 to 1.51)	⊕⊕◯◯ Low	

DBP SALT 8 week follow up.

2	RCT	Not Serious	Very serious	Not serious	Serious	None	60	58	-	-1.08 (-1.54 to -0.62)	⊕⊕◯◯ Low	
